# Effect of Compatibilizer on the Persistent Luminescence of Polypropylene/Strontium Aluminate Composites

**DOI:** 10.3390/polym14091711

**Published:** 2022-04-22

**Authors:** Anesh Manjaly Poulose, Hamid Shaikh, Arfat Anis, Abdullah Alhamidi, Nadavala Siva Kumar, Ahmed Yagoub Elnour, Saeed M. Al-Zahrani

**Affiliations:** 1 SABIC Polymer Research Center, Department of Chemical Engineering, King Saud University, Riyadh 11421, Saudi Arabia; hamshaikh@ksu.edu.sa (H.S.); aarfat@ksu.edu.sa (A.A.); akfhk90@hotmail.com (A.A.); szahrani@ksu.edu.sa (S.M.A.-Z.); 2 Department of Chemical Engineering, King Saud University, Riyadh 11421, Saudi Arabia; snadavala@ksu.edu.sa (N.S.K.); aelnour@ksu.edu.sa (A.Y.E.)

**Keywords:** phosphorescent composites, compatibilizer, thermal and mechanical

## Abstract

There is a demand for long afterglow composites due to their potential applications in nighttime signal boards, sensors, and biomedical areas. In this study, Polypropylene (PP)/strontium aluminate-based composites [SrAl_2_O_4_:Eu^2+^/Dy^3+^ (SAO_1_) and Sr_4_Al_14_O_25_: Eu^+2^, Dy^+3^ (SAO_2_)] with maleic anhydride grafted PP compatibilizer (PRIEX) were prepared, and their auto-glowing properties were examined. After UV excitation at 320 nm, the PP/5PRIEX/SAO_1_ composites showed green emission at 520 nm, and blue emission was observed for PP/5PRIEX/SAO_2_ around 495 nm. The intensity of phosphorescence emission and phosphorescence decay was found to be proportional to the filler content (SAO_1_ and SAO_2_). The FTIR analysis excluded the copolymerization reaction between the SAO_1_ and SAO_2_ fillers and the PP matrix during the high-temperature melt mixing process. The SAO_1_ and SAO_2_ fillers decreased the overall crystallinity of the composites without affecting the T_m_ and T_c_ (melting and crystallization temperature) values. The thermal stability of the composites was slightly improved with the SAO_1_ and SAO_2_ fillers, as seen from the TGA curve. Due to the plasticizing effect of the compatibilizer and the agglomeration of the SAO_1_ and SAO_2_ fillers, the tensile modulus, tensile strength, and storage modulus of the composites was found to be decreased with an increase in the SAO_1_ and SAO_2_ content. The decreasing effect was more pronounced, especially with the bulk-sized SAO_2_ filler.

## 1. Introduction

Strontium aluminates doped with rare earth metals can store energy once excited by UV, thermal, or mechanical stimulation [1,2,3,4,5]. These materials show persistent luminescence once the excitation energy is released as light in the visible wavelength range, especially during nighttime. Unlike ZnS, these materials have good chemical stability, are non-radioactive, and have long-lasting afterglow properties (≥16 h) even after the excitation source is stopped [6]. They find potential applications in emergency signs, medical diagnostics, luminous paints, optical detectors, textiles, etc. [7,8,9,10,11,12]. After the discovery of green phosphor, SrAl_2_O_4_:Eu^2+^/Dy^3+^, in the 1990s, the need for multi-colored persistent phosphor encouraged rapid growth in this area [13]. In recent years, different rare-earth such as Pr^3+^, Ce^3+^, Sm^2+^, Nd^3^ doped luminescent materials were developed, and their emission colors were dependent upon the raw materials chosen, stoichiometry, the synthesis methods, and their crystalline structure [14,15,16]. Due to the shorter afterglow time and lower luminescent intensity, these were not suited for practical application purposes. So far, mainly SrAl_2_O_4_: Eu, Dy, and Sr_4_Al_14_O_25_: Eu, Dy phosphors were successfully commercialized due to their superior afterglow characteristics. Lephoto et al. compared the effect of doping different trivalent rare-earth (Re^3+^, Dy^3+^, Nd^3+^, Gd^3+^, Sm^3+^, Ce^3+^, Er^3+^, Pr^3+^, and Tb^3+^) on BaAl_2_O_4_:Eu^2+^ phosphors and found that the highest intensity was obtained for the Er^3+^ and Dy^+3^ doped ones. When comparing both the luminescence intensity and decay effect, the Dy^+3^ doped ones showed better luminescence intensity as well as slow decaying [17]. 

For the long-term application side, these luminescent particles need to be combined with the polymer matrix to prevent hydrolysis in the presence of humidity, and the polymeric encapsulation method yields good mechanical strength, support, and flexibility to these composites [18]. When the polymer organic moieties and inorganic phosphor materials are physically mixed, they often deteriorate the mechanical properties due to the weaker interaction between the polymer and filler (immiscibility). Thus, designing a phosphors/polymer composite with good physical properties is still challenging. Furthermore, the melt-blending of phosphor fine powder with polymers often results in an agglomeration effect inversely affecting the physical properties of the composites. In the literature, different polymers were incorporated with luminescent materials to facilitate better adhesion and support [19]. Polyurethane (PU) with amino-functionalized SrAl_2_O_4_:Eu^2+^, Dy^3+^ exhibits good compatibility between the phosphor and PU and imparts better mechanical properties than non-functionalized phosphor/PU composites [20]. Bem et al. employed LDPE and PMMA as polymer support for the SrAl_2_O_4_:Eu, Dy phosphor, and the results show that the LDPE/phosphor has higher luminous intensity as it forms a three-dimensional phosphor network in the LDPE matrix [21]. In a recent study, SiO_2_ modified SrAl_2_O_4_:Eu^2+^, Dy^3+^ showed better dispersion in PLA and PMMA and improved the mechanical properties of the composites with better antibacterial properties and hydrophobicity [19,22]. In another study, SrAl_2_O_4_:Eu^2+^, Dy^3+^ phosphor with TiO_2_ in the PMMA matrix considerably improved the luminescence emission intensity. The TiO_2_ nanoparticles act as a light-harvesting agent enriching the light absorption capacity of the phosphor [23]. Recently, PP/SrAl_2_O_4_:Eu^2+^, Dy^3+^ long afterglow composites were prepared and studied. It was found that the resultant composites showed long afterglow properties, but their tensile strength and modulus decreased significantly [24]. PEG plasticizer was also added, aiming to improve the dispersion of the phosphor, but it inversely affected the intensity of emission [24]. In this paper, maleic anhydride grafted PP was added to virgin PP and melt-blended with SrAl_2_O_4_:Eu^2+^, Dy^3+,^ and Sr_4_Al_14_O_25_: Eu^2+^, Dy^3+^, aiming to improve the compatibility of the phosphor with the PP matrix and thereby on the mechanical features of the resulting long-lasting luminescent composites. 

## 2. Materials and Methods

### 2.1. Materials

Poly(propylene) (PP) matrix, supplied by TASNEE Company, Saudi Arabia (Grade: PP H4120), was chosen for the present study. PP with a density of 0.9 g/cm^3^ and a melt flow rate (MFR) of 12 g/10 min was employed as the polymer matrix. Two strontium aluminate phosphor materials; SrAl_2_O_4_: Eu^+2^, Dy^+3^ (Mw = 209.11 g/mol) (SAO_1_), and Sr_4_Al_14_O_25_: Eu^+2^, Dy^+3^ (1139.55 g/mol) (SAO_2_) from Sigma Aldrich company were used as luminescent fillers. Maleic Anhydride Grafted PP homopolymer (PRIEX 20097; Addcomp polymer additive solutions, Nijverdal, Netherlands) was used as a compatibilizer with an MFR of 20–30 g/10 min.

### 2.2. Methods

#### 2.2.1. Preparation of the Composites 

PP pellets and phosphor powders (SAO_1_ and SAO_2_; 1, 3, 5, and 10 wt. %) were mixed physically before feeding into the melt-mixing chamber. The PRIEX compatibilizer weight percentage was fixed as 5 for the entire study. The DSM Xplore micro-compounder (15cc) (Sittard, The Netherlands) with a co-rotating twin-screw was utilized for the melt-mixing process. The melt-mixing process was carried out for 3 min at a temperature of 200 °C (screw speed of 50 rpm). The collected melt was then introduced to an injection-molding machine (DSM Xplore microinjection molder, 12cc, Sittard, The Netherlands) for the preparation of the ASTM, Type1 tensile testing specimen. Thin films of a thickness (0.6 cm) for the phosphorescence measurements were prepared using COLLIN Press (Maitenbeth, Germany) by applying 100 bar pressure at 200 °C. 

#### 2.2.2. Characterization of Composites

##### Phosphorescence and Decay Measurements

A Fluorescence Spectrophotometer (Agilent Technologies, Santa Clara, CA, USA) was used to measure the phosphorescence in the prepared composites. The emission spectra (visible range) were collected after exciting the samples using a UV source at a wavelength of 320 nm. The phosphorescence decay studies were conducted in this machine with the gate time and delay time set as 10,000 ms and 0.1 ms. The emission intensity decay was examined in 1800 s, selecting the excitation and emission wavelength as 320 nm and 490 nm, respectively. 

##### Scanning Electron Microscope (SEM)

The phosphor dispersion and the morphology of the PP-5PRIEX**/**SAO_1_ and PP-5PRIEX/SAO_2_ composites were monitored with the help of the SEM (VEGA II LSU, TESCAN, Libusina, Czech Republic) at an accelerating voltage of 10 kV. The thin-sliced specimens were placed on sample holder stubs using double-sided adhesive carbon tape and were then coated with a fine layer of gold for 40 s to eliminate the charging effect.

##### Fourier Transform Infrared Spectroscopy (FTIR)

Fourier transform infrared spectroscopy (ATR-FTIR) analysis was carried out for the composites using a Nicolet iN10 FTIR microscope (Thermo-Scientific, Winsford, UK) with a Germanium micro tip accessory, and the scanning range was between 400 and 4000 cm**^−^**^1^ wavelengths. 

##### Differential Scanning Calorimetry (DSC)

The DSC-60A model (Shimadzu, Tokyo, Japan) was operated for the thermal characterization of the studied composites. The composites were heated from 30 to 250 °C with a heating rate of 10 °C/min. At 250 °C, the samples were kept for 4 min to erase the memory effect and were then cooled to 30 °C at the same rate. The degree of crystallinity ($X_{c}$) was calculated using the following equation:

(1) $$X_{c}=\frac{\left(\Delta H_{m}\right)}{\left(1-\emptyset \right)\Delta H_{m}^{o}}\times 100$$

where $\Delta H_{m}$ is the melting enthalpy, and $\Delta H_{m}^{o}$ is the enthalpy of melting for a 100% crystalline PP which is 207 J/g [25]. ∅ is the phosphor content (wt. %) present in the composites.

##### Thermo-Gravimetric Analysis (TGA)

The thermal stability of the PP-5PRIEX**/**SAO_1_ and PP-5PRIEX**/**SAO_2_ composites was investigated as a function of phosphors contents using a thermo-gravimetric analyzer, Mettler Toledo AG, Analytical CH-8603, Schwerzenbach, Switzerland. The composites (9–11 mg) were scanned in an aluminium pan from 30 to 700 °C, at a heating rate of 10° C/min under an inert Argon gas flow rate of 50 mL/min. The weight loss against the temperature was monitored.

##### Tensile Testing

Tensile tests were carried out in a universal testing machine (UTM) (Tinius-Olsen, Horsham, Pennsylvania, USA, Model: H100KS). The test was conducted according to the ASTM D638-14 testing method for the tensile properties of plastics. The reported values are an average of five measurements. 

## 3. Results

### 3.1. FTIR, DSC, and TGA Studies in PP/PRIEX/SAO_1_ and PP/PRIEX/SAO_2_ Composites

**Figure 1:**
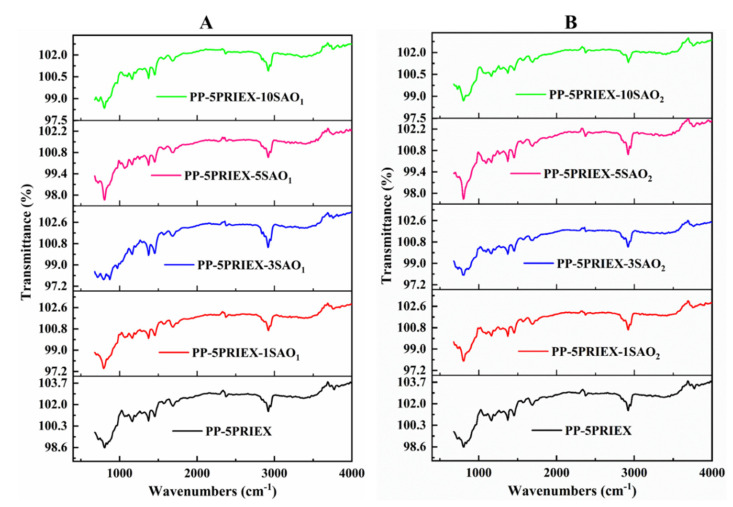
ATR-FTIR data of PP/PRIEX/SAO_1_ (**A**) and PP/PRIEX/SAO_2_ (**B**) composites.

The ATR-FTIR data of PP/PRIEX**/**SAO_1_ and PP/PRIEX**/**SAO_2_ composites are shown in Figure 1. For all the composites studied, the FTIR peaks were identical, and one can say that the chemical reaction between the phosphor particles SAO_1_ and SAO_2_ and the PP/PRIEX matrix did not take place during the high-temperature mixing process. As there is no new peak formation visible in the FTIR spectrum of the composites, only physical mixing was taking place during the high-temperature processing of all the composites.

The DSC data for the SAO_1_ and SAO_2_ composites are presented in Table 1 and Table 2, respectively. The SAO_1_ and SAO_2_ fillers did not affect the melting and crystalline temperature (T_m_ and T_c_) of the composites, which is also evidence of the physical mixing process. The chemical reaction altered the T_m_ and T_c_ values due to the structural modification taking place during the reaction, and in turn, it affected the composite’s crystallization process [26]. As seen in Table 1 and Table 2, the SAO_1_ and SAO_2_ fillers decreased the overall crystallinity of the resultant composites. The reason was due to the SAO_1_ and SAO_2_ fillers interception, which restricted the PP chain mobility, hindered the chain packing and spherulites formation, and decreased the overall crystallinity of the composites [27]. 

**Figure 2:**
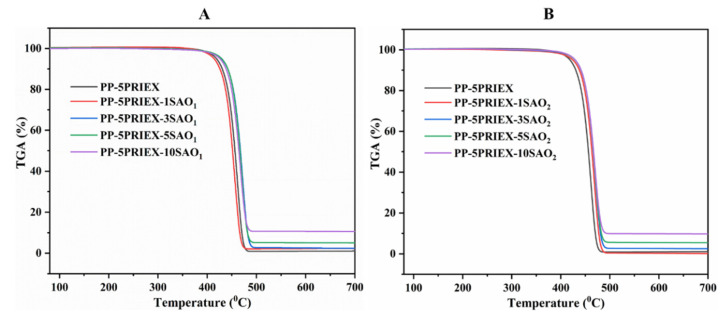
TGA results of PP/PRIEX**/**SAO_1_ (**A**) and PP/PRIEX**/**SAO_2_ (**B**) composites.

The TGA data for the PP/PRIEX**/**SAO_1_ and PP/PRIEX**/**SAO_2_ composites are in Figure 2A,B. For all the composites studied, the degradation process was taking place in a single step. The inorganic fillers (SAO_1_ and SAO_2_) which act as thermal barriers, slightly improved the thermal stability of the studied composites [22]. The onset of degradation was slightly shifted towards a higher temperature as seen in Figure 2. The SAO_1_ and SAO_2_ fillers are thermally stable inorganic materials and the residues left over after heating was directly proportional to the amount of SAO_1_ and SAO_2_ fillers used. 

### 3.2. Tensile Strength, Tensile Modulus and Storage Modulus of PP/PRIEX/SAO_1_ and PP/PRIEX/SAO_2_ Composites

**Figure 3:**
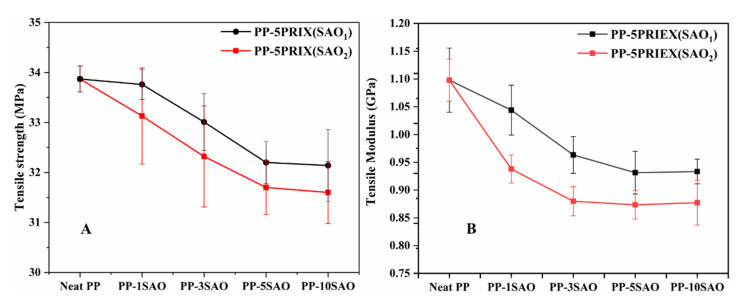
The tensile strength (**A**) and tensile modulus (**B**) of PP/PRIEX**/**SAO_1_ and PP/PRIEX**/**SAO_2_.

**Figure 4:**
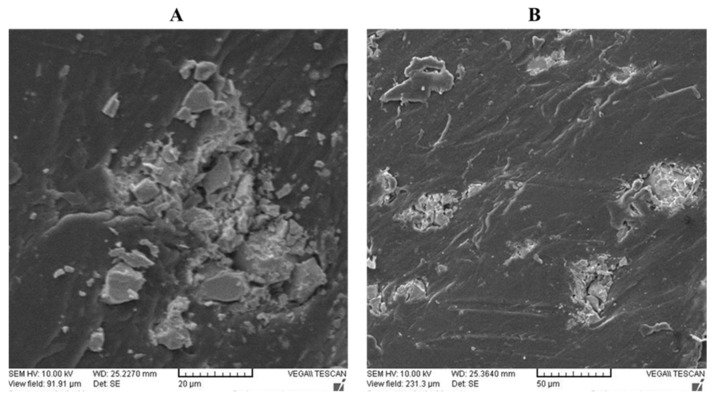
The SEM pictures were taken on PP/PRIEX/10 SAO_1_ (**A**) and PP/PRIEX/10SAO_2_ (**B**).

The tensile strength (TS) and tensile modulus (TM) of the PP/PRIEX/SAO_1_ and PP/PRIEX**/**SAO_2_ composites are shown in Figure 3A,B. Both TS and TM decrease with an increase in the weight percentage of SAO_1_ and SAO_2_ in the studied composites. The TS decreases from 34 MPa to 32.1 for SAO_1_ composites and 34 to 31.6 for SAO_2_ composites with the highest filler loading (10 wt. %). For the highest filler loading, the TM decreases from 1.1 to 0.93 GPa for SAO_1_ and 1.1 to 0.88 GPa for SAO_2_ composites. Due to the presence of the PRIEX compatibilizer, the decrease in the tensile properties (TS and TM) is not very pronounced, and the agglomeration of SAO_1_ and SAO_2_ exists, as seen in Figure 4, which could be the reason for the existing decreasing effect. The incompatibility among the inorganic fillers (SAO_1_ and SAO_2_) with the organic polymeric moieties can also impart mechanical property deterioration [24]. 

**Figure 5:**
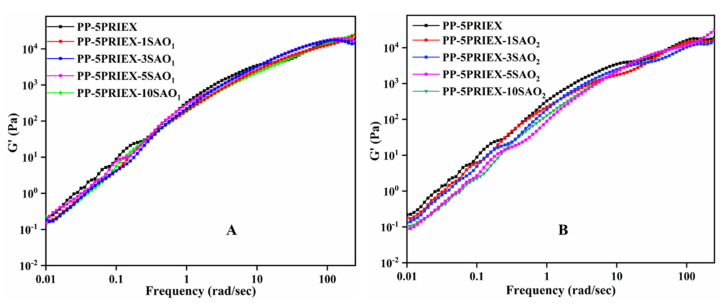
The storage modulus of PP/PRIEX**/**SAO_1_ (**A**) and PP/PRIEX**/**SAO_2_ (**B**) composites.

The storage modulus of the PP/PRIEX/SAO_1_ and PP/PRIEX/SAO_2_ composites from the rheological studies are shown in Figure 5. The rheological properties also follow the tensile properties trend, i.e.**,** a slight decrease in storage modules exists, which could be due to the agglomeration of SAO_1_ and SAO_2_ fillers in PP (Figure 4). 

### 3.3. Phosphorescence Emission and Their Decay Studies in PP/PRIEX/SAO_1_ and PP/PRIEX/SAO_2_ Composites

**Figure 6:**
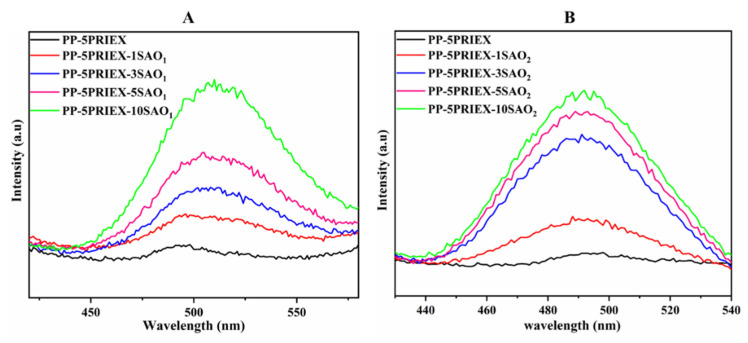
The phosphorescence emission spectra of PP/5PRIEX/SAO_1_ (**A**) and PP/5PRIEX**/**SAO_2_ (**B**) composites.

**Figure 7:**
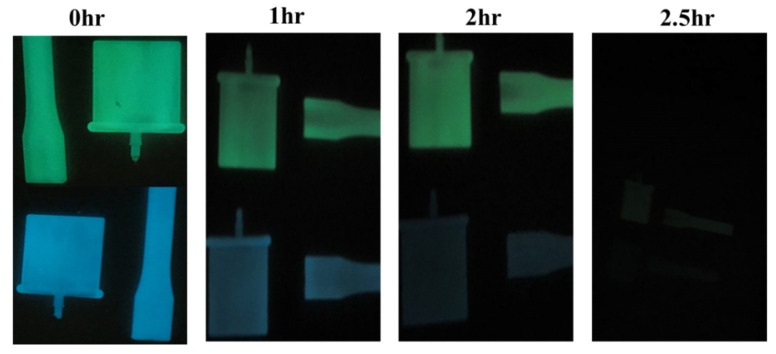
The green and blue emission decay at different times for PP/5PRIEX/10SAO_1_ and blue PP/5PRIEX/10 SAO_2_ composites (after excited in outside sunlight for 10 min and kept in a dark room).

The phosphorescence emission spectra of PP/5PRIEX/SAO_1_ and PP/5PRIEX/SAO_2_ were collected after exciting the composites at an excitation wavelength of 320 nm (UV). The emission spectra of both the composites are shown in Figure 6A,B. The emission spectra of the PP/5PRIEX/SAO_1_ composites showed a green emission at 520 nm, and for PP/5PRIEX/SAO_2_, a blue emission was observed around 495 nm and the intensity of the emission increased with the SAO_1_ and SAO_2_ phosphor content. The reported mechanisms for the phosphorescence emission are controversial [13,28,29], but it is widely accepted that Eu^2+^ luminescence center transition (5d–4f) is responsible for the broadband at the visible wavelength range. Later, Yuan et al. suggested the important role of Dy^+3^ in the long-lasting afterglow process. They suggested that these Dy^3+^ ions act as luminescence centers and introduce new electron traps and significantly increase the concentration of electron or hole traps. The electrons can be excited from the valence band (VB) to the conduction band (CB), creating excited electrons and holes. These can be non-radiatively captured by the electron or hole traps by the quantum tunneling process known as the trapping process. Once the trapped electrons and holes are released from their traps, they combine radiatively with the result of afterglow, and the depths of these electron and hole traps are critically important for this process [30,31]. For the PP/5PRIEX/10SAO_1_ and PP/5PRIEX/10SAO_2_ samples, once they were excited in outside sunlight for 10 min and kept under darkness, the phosphorescence emission was found to last for above 2 h, as shown in Figure 7. 

**Figure 8:**
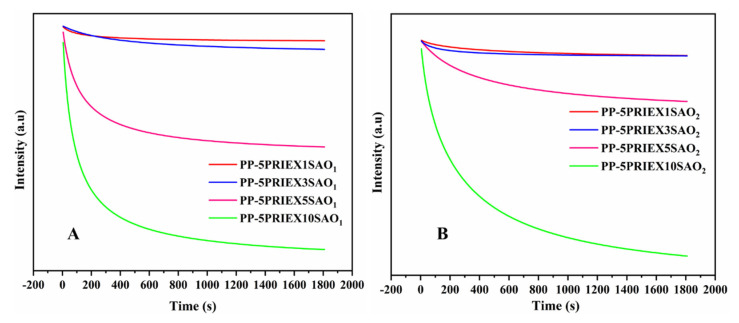
The phosphorescence intensity decay with time for PP/PRIEX/SAO_1_ (**A**) and PP/PRIEX/SAO_2_ (**B**) composites.

The phosphorescence intensity decay with time for the studied composites is displayed in Figure 8. The decay rate was found to be directly proportional to the filler (SAO_1_ and SAO_2_) content in the studied PP/PREX matrix. With the gradual increase of the SAO concentration, the initial afterglow intensity gradually decreased, as shown in Figure 8. This is probably because one part of the excitation energy was absorbed and reflected by the host PP and PRIEX interaction with the rare-earth-doped particles. The part of the energy emitted by the SAO phosphors was also absorbed and reflected by the host matrix [32]. As shown in Figure 8, the phosphorescence decay curve shows two distinct behaviors, initially a faster decay followed by slow decay. Once the excitation of the low trap energy level was completed, the thermal disturbance initiated the release of the electrons. The low trap level had a shallow depth, low energy, and small binding effects on the electrons. Therefore, the reason for the faster decay of the original brightness could be related to the faster escape of the electrons from the low trap energy level [33]. The final intensity did not reach zero within the period used for the decay studies, as the afterglow properties lasted for hours in these composites [34].

## 4. Conclusions

PP/Maleated PP encapsulated strontium aluminate fillers (SAO_1_ and SAO_2_) with long-lasting afterglow properties were prepared via the melt-blending process. The intention of using maleated PP was to improve the dispersion of SAO_1_ and SAO_2_ fillers in the PP matrix and thereby have a positive effect on the mechanical properties of the resultant composites. As visible from the SEM figures, the agglomeration of these fine SAO_1_ and SAO_2_ fillers and the incompatibility among fillers and matrix inversely affected the mechanical properties (tensile strength, tensile modulus, and storage modulus) of the resultant composites, especially with the bulk-sized SAO_2_ filler. Though chemically modified maleated PP was used in this study, no chemical reaction took place between the SAO_1_ and SAO_2_ fillers and the PP matrix. This was confirmed from the FTIR and DSC studies, and from TGA studies, it was found that the onset of degradation was slightly shifted to a higher temperature with the filler percentage. The fillers inversely affected the total crystallinity of the composites without changing the T_m_ and T_c_ values. As expected, the intensity of phosphorescence emission and their decay rate were enhanced with the phosphor filler content. Though economically viable long-lasting composites based on PP and maleated PP were fabricated, the efforts must be continued to improve the dispersion of phosphor fillers and, thereby, the mechanical properties of the composites. 

## Figures and Tables

**Table 1:** DSC data on PP/5PRIEX**/**SAO_1_ composites.

Material	T_c_ (°C)	T_m_ (°C)	Δ*H_m_* (J/g)	*X_c_* (%)
PP-5PRIEX	116.1	158.8	99.4	48.0
PP-5PRIEX/1SAO_1_	115.1	160.2	87.9	42.5
PP-5PRIEX/3SAO_1_	114.0	160.8	70.5	34.1
PP-5PRIEX/5SAO_1_	114.8	159.2	67.6	32.7
PP-5PRIEX/10SAO_1_	114.4	160.1	62.1	30.0

**Table 2:** DSC data on PP/5PRIEX**/**SAO_2_ composites.

Material	T_c_ (°C)	T_m_ (°C)	Δ*H_m_* (J/g)	*X_c_* (%)
PP-5PRIEX	116.1	158.8	99.4	48.0
PP-5PRIEX/1SAO_2_	114.5	160.2	81.2	39.2
PP-5PRIEX/3SAO_2_	115.4	159.8	79.9	38.6
PP-5PRIEX/5SAO_2_	115.3	159.6	75.6	36.5
PP-5PRIEX/10SAO_2_	115.8	159.1	72.1	34.8

## Data Availability

The data presented in this study are available on request from the corresponding author.
